# Clinical Cases in Medical Wards of Mercy Hospital

**Published:** 1871-04

**Authors:** 


### Clinical Reports.

### CLINICAL CASES IN MEDICAL WARDS OF MERCY HOSPITAL.

Clinic by PROF. DAVIS. From Notes by S.

CASE I.—This girl was taken sick last Thursday; admitted to the hospital yesterday, Monday, January 2d.

She presents the characteristic eruption of measles; easily distinguished from that occurring in scarlatina, by the points of eruption being aggregated in clusters, with natural skin between, and from that of small-pox, in which the eruption comes in pointed, elevated, hard nubes, not so red as in this case, presenting only a slight flush, and without the catarrhal premonitory symptoms, or weeping of the eyes.

The patient is now at the stage when she is suffering oppression and tightness in the chest, with a harsh cough, which produces intense pain through the temples with each paroxysm.

TREATMENT.—In this disease, if uncomplicated, the indications are very plain and simple. The disease has a natural course to run; cannot be broken up; is self-limited; and the physician is not expected to interfere with active means, but simply to modify its severity as much as possible, so as to leave the system in the best possible condition.

Give enough of some anodyne expectorant to lessen the severity of the bronchial irritation and cough, and mitigate the pain in the head.

One of the best preparations, perhaps, for cases as severe as this, consists in the following:

℞. Syrup Scilla Comp.----------------------℥iss.
Vinum Antimonii,------------------------℥ss.
Tinct. Opii Camp.,-----------------------℥ij.
Tinct. Verat. Viride,---------------------ʒi.

M. Dose—one teaspoonful every three hours in a tablespoonful of water.

This will, usually, in the course of twenty-four hours, lessen the fever and modify the cough, while the pain in the head will at the same time be greatly relieved.

As the period is reached when the fever begins to decline, the veratrum may be omitted from the mixture, which may then be continued, given every three, four, or five hours, till the cough has entirely disappeared; the anodyne with the expectorant, produces a very pleasant effect, and does not tend to interfere with the progress of the eruption; but you will usually find while using this, that the bowels will become constipated, tongue coated, urine scanty, etc., which condition, if neglected, leads to a bad state of digestion, disorder of the bowels, etc.

I am not in the habit of giving physic until the eruption is fairly out; when, if the bowels have not moved for a couple of days, I direct a mild laxtive, as, for instance, a combination like the following:

℞. Hydrarg. Chl. M.,--------------------grs. v.

Leptandrin,-----------------------------grs. ij.

Soda Bicarb.,---------------------------grs. v. M.

This will produce a moderately fair operation of the bowels,

14

which may subsequently be kept in a regular condition by any simple laxative that will be most readily taken. Rochelle salts, effervescing solution, Tanant's aperient, pil rhei comp., etc.

You will occasionally meet with cases where the patient suffers so much at night that it is best to give a tolerably full dose of Dover's powder at bedtime; a good combination for this purpose is, pulv. Dover, viii grs. with hydrag. chl. mit. i gr., followed, if necessary, by a laxative in the morning. Or, you may use instead, fifteen or twenty grains of the bromide of potassium at night; this sometimes acts very favorably, but is not reliable in cases of eruptive fever.

One important thing to guard against, is, extension of the irritation from the bronchial tubes, to the lobules of the lungs; making it complicated with lobular pneumonia.

In children under two years of age this tendency is very strong.

It is usually about the second day of the eruption that you will first be able to detect this; if it is going to occur, it may happen later, seldom, however, before the second or third day.

You will observe, first, that they do not breathe naturally, nor as from simple tightness of the bronchial tubes, but every inspiration brings out a forcible expansion of the nostrils, and at the beginning of the respiratory act, there is sudden falling in of the walls of the abdomen, produced by contraction of the abdominal muscles.

These, if the case be noticed carefully, will be among the earliest symptoms to warn you of trouble with the lungs. On applying the ear to the chest, you will also find a sharp, well-defined, subcrepitant ronchus.

If allowed to run along till the time for the fever to subside, you will have the little lobules of the lungs in a hepatized condition, the patient drowsy and dull, breathing short, lips blue, pulse sharp, short, and quick in stroke, and easily compressed, lips and tongue dry, patient disposed to lay with the head thrown back, eyes half open, and in a few days death will supervene.

In adults, this is the chief source of fatality from measles.

When symptoms of pneumonia occur, in connection with measles, the best remedy in children is a combination like the following:

℞. Liq. Ammon. Acetas,----------------------℥iss.
Syrup Ipecac,------------------------------℥ss.
Tinct. Opii Camp.,-----------------------℥i.
Tinct. Verat. Viride,----------------------ʒi.

Dose—proportioned to the age of the child; for a child two years old, about twenty drops are usually necessary; though it is best to begin with ten drops; for an adult one teaspoonful. It should be given every two, three, or four hours, till the fever is controlled. In the active stage of the disease, while using this mixture, cover the chest externally with fomentations.

I have considerable faith in the popular notion about onions; they certainly afford more relief to the breathing than any other thing we can use. I attribute it to the impregnation of the air which is inhaled, with the volatile oil, more than to any absorption from the surface of the chest, and think this application preferable to blistering.

It will be advisable, in these cases, to give a powder, containing from half a grain to a grain of calomel, with Dover's powder, according to the age and restlessness of the patient, about three times a day. The liquid mixture may be continued, at longer intervals, until the symptoms of pneumonia have entirely disappeared.

Case 2.—General Dropsy, etc.—December 6th, A. B., age —, occupation ————, etc. This patient came into the hospital several days ago, presenting the appearance of general dropsy, with great dyspnœa and swelling of the lower extremities.

It seemed that he had had several attacks of bronchial irritation, and asthmatic restriction of the bronchial tubes, with congestion of the capillaries of the tubes, and irritation of the pneumogastric nerve, causing great difficulty of breathing, imperfect æration of the blood, tightness of the chest, and more or less harsh, suffocating cough. With this condition of the bronchial tubes he had decided pain in the left side of the chest, lips blue, face bloated, legs and feet very much swollen, and a general infiltration of the tissues, scrotum swollen, full, and affected with an eczenatous eruption, accompanied with a burning, smarting, tormenting sensation, dulness over a larger space than normal in the cardiac region, and abdomen distended from the effusion.

On application of the stethoscope, find heart sounds distant and obscure, with hardly distinguishable impulse, and am unable to make any distinction between the first and second sounds, heart has a tremendous rapid motion. This, with excessive dulness, indicating pericardial effusion.

The first impression would be that we had albuminuria or organic disease of the kidney, giving rise to impoverishment of the blood, inducing dropsical effusion into the cavity of the pleura-pericadium, etc., that so frequently supervenes during the progress of albuminuria, but upon examination of the urine this could not be detected.

Not being able to account for the effusion in this way, we might expect to find some enlargement and obstruction to the circulation in the abdominal viscera, as enlargement of the liver in persons who drink habitually, or the formation of fatty deposit in the liver, or in the muscular structure of the heart, which would give rise to dropsical effusion, and in such a case the abdomen would become greatly distended, and it would be some time before it pervades the extremities; the same is true of the spleen. If the liver was enlarged we could easily bring the fingers in contact with the enlarged organ just below the margin of the ribs, and the same in regard to the spleen—also, by percussion.

In this case, instead of dulness below the margin of the ribs, this is the most resonant part of the abdomen, probably owing to the fact that the colon is distended with gas, in which case, however, if the organs above were distended, it would be crowded down or overlapped, and we should get dulness on percussion; if from the spleen, the dull region describing a curve on the left side.

If not albuminuria, enlargement of these organs, etc., how shall we account for the symptoms?

If true that it began with an attack of capillary bronchitis, in conjunction with pericardial irritation, continued so as to retard decarbonization of the blood, with sufficient pericardial effusion to embarrass the heart's action, combining to induce impairment of the vaso-motor nervous system, this might give rise to a loss of tone and relaxation of the capillaries, allowing a slow circulation of the blood through them, and exomoiosis from a want of impulse, and reaction under the influence of the vaso-motor system of nerves upon the muscular fibres of the arterial system. The blood does not get more than one-half the normal amount of oxygen, and this condition acts as a sedative narcotic on the whole capillary system.

As I cannot determine the existence of any disease in the viscera of the abdomen that would account for this condition—though it is not usual to get dropsy so rapidly from obstruction in the air-passages, etc.—as I could find no other cause, and from the weak character of the pulse, blue lip, and general relaxation, as manifested by the effusion, I should be led to conclude that such was the case in the present instance.

There was, in the beginning, that stage that would admit of active antiphlogistic treatment, when, I have no doubt, a sedative and alterative course of treatment would have arrested its progress, but the time for arterial sedatives has passed away, we now have too feeble capillary circulation, and a weak, tumultuous action of the heart.

The object in the treatment should be to give more force to the heart's contractions, reducing the frequency at the same time, and to produce efficient action of the kidneys, to carry off the water and prevent further effusion.

To accomplish this we relied upon the digitalis and scutillaria to control the force and frequency of the heart's action, while the influence of the digitalis on the kidneys makes it very applicable in this case.

We prescribed the following mixture:

℞. Fl. Ext. Scutillaria,------------------------℥ij.

" " Digitalis,---------------------------℥i.

" " Hyoscyamus,----------------------℥i.

Potas. Nitras,------------------------------℥iij. M.

S. One teaspoonful to be taken every three hours.

For a future alterative influence I directed, in addition, a powder consisting of Dover, vi grs. hydrag. chl. mit., ii grs., to be given every night. Doubt if the chloride had better be continued more than another day.

If at any time the bowels should become so loose as to weaken the patient, would reduce the frequency of the liquid mixture to once in four hours, and alternate with it a teaspoonful of the ordinary turpentine and laudanum emulsion.

Will also have a blister applied over the chest, followed by poultice.

Under the above course of treatment the dropsical effusion and difficult breathing were rapidly relieved, and on the third day the pulse became slower and more distinct, and on the fifth day it was so slow that the digitalis had to be diminished by lengthening the interval from once in three hours to every six hours; cough easier, but dry, so that we gave the muriate ammonia between the other. Makes water quite freely, breathing tolerably fair, but still indicative of bronchial tightness, and the heart's action, while slow, is irregular, impulse still deficient and hardly distinguishable.

First and second sounds of the heart cannot be made out clearly, and are distant still.

This feeble and irregular action of the heart may arise in three conditions, one source is pericardial effusion, another is failure of innervation, and thirdly, as remarked at the previous clinic, by organic change in the muscular structure of the heart itself. When either are strongly developed, we may have the heart's action so weakened and imperfect that it is impossible to make any distinction between the first and second sounds, and if the patient is not under some influence to regulate the heart's action, it will beat with great rapidity, especially in degeneration and effusion. Occasionally, in defective innervation, the action may be slow and weak, but this is a rare occurrence. The health-beat generally increases in frequency with diminished force.

The improvement in the effusion and oxidation of the blood while the heart remains feeble, increases the probability that we may have impairment of the muscular structure of the heart by fatty degeneration.

Under this supposition, and with a view to counteracting the formation of fat by increasing the oxidization of the carbonaceous fatty matters, and improving the tendency to nitrogenous nutrition, I shall direct potas. chloral, x grs., in mucilage of gum Arabic, after each meal; giving the ammonia mixture night and morning, and the digitalis mixture every six hours.

